# Dissecting the dynamics of dysregulation of cellular processes in mouse mammary gland tumor

**DOI:** 10.1186/1471-2164-10-601

**Published:** 2009-12-13

**Authors:** Wieslawa I Mentzen, Matteo Floris, Alberto de la Fuente

**Affiliations:** 1 CRS4 Bioinformatica, Parco Scientifico e Technologico POLARIS, 09010 Pula (CA), Italy

## Abstract

**Background:**

Elucidating the sequence of molecular events underlying breast cancer formation is of enormous value for understanding this disease and for design of an effective treatment. Gene expression measurements have enabled the study of transcriptome-wide changes involved in tumorigenesis. This usually occurs through identification of differentially expressed genes or pathways.

**Results:**

We propose a novel approach that is able to delineate new cancer-related cellular processes and the nature of their involvement in tumorigenesis. First, we define modules as densely interconnected and functionally enriched areas of a Protein Interaction Network. Second, 'differential expression' and 'differential co-expression' analyses are applied to the genes in these network modules, allowing for identification of processes that are up- or down-regulated, as well as processes disrupted (low co-expression) or invoked (high co-expression) in different tumor stages. Finally, we propose a strategy to identify regulatory miRNAs potentially responsible for the observed changes in module activities. We demonstrate the potential of this analysis on expression data from a mouse model of mammary gland tumor, monitored over three stages of tumorigenesis. Network modules enriched in adhesion and metabolic processes were found to be inactivated in tumor cells through the combination of dysregulation and down-regulation, whereas the activation of the integrin complex and immune system response modules is achieved through increased co-regulation and up-regulation. Additionally, we confirmed a known miRNA involved in mammary gland tumorigenesis, and present several new candidates for this function.

**Conclusions:**

Understanding complex diseases requires studying them by integrative approaches that combine data sources and different analysis methods. The integration of methods and data sources proposed here yields a sensitive tool, able to pinpoint new processes with a role in cancer, dissect modulation of their activity and detect the varying assignments of genes to functional modules over the course of a disease.

## Background

Breast cancer is a heterogeneous disease, both with respect to cells of origin and the underlying course on the molecular level [1]. Variable series of cellular events may lead to the formation of malignancy, but to date the nature and sequence of many of the processes that go awry during tumorigenesis remain elusive. The value of such knowledge cannot be overestimated for understanding the disease and outlining the effective treatment.

With this aim in mind, we devised a novel bioinformatics approach, taking advantage of the abundance of available functional genomics data. Integration of heterogeneous data allows extraction of knowledge that is not evident when examining data of different types separately and provides a holistic view on the functioning of the biological system on multiple levels [2,3]. Our approach goes beyond traditional microarray analysis, because it considers Protein Interaction Network modules as gene groups in a joint differential expression-differential coexpression analysis.

First, we use Protein Interaction Network (PIN) modules to delineate biological processes as an alternative to 'textbook pathways'. Several approaches for partitioning a bio-molecular network of interactions into sensible and coherent functional units have been proposed [4,5]. Here we define modules as the densely interconnected regions in the PIN, i.e., groups of proteins in the network that are distinguishable from the neighborhood due to a much higher density of interactions among them than with other proteins in the network. Proteins heavily interconnected by a network of mutual interactions are likely to be involved in the same biological process [6-8]. Such topology-based designation of modules is not constrained by existing annotation of pathways, and allows finding novel disease-specific modules. This network-guided approach is especially useful in the study of cancer, since this disease proceeds through step-wise accumulation of defects in biological processes, whose nature is often not known [9]. In constantly evolving cancerous cells, signaling and metabolic pathways might be disrupted or modified to better serve the cells' needs and particular genes might trade their usual housekeeping function for a different one; some natural control mechanisms might be turned off, while others could be triggered. These events can be reflected in changes of the level and cohesiveness of gene expression profiles of affected processes. Therefore we subjected the network modules to differential expression and differential coexpression tests, in search of not only the processes that alter their intensity, but also the ones whose degree of coregulation differs between the disease stages.

The identification of network elements whose altered activity is associated with disease has been pursued in several recent studies. Choi et al. (2005) constructed coexpression networks specific for cancer and normal tissue and identified pairs of Gene Ontology categories most often represented by the pairs of differentially coexpressed genes [10]. A similar approach was adopted by Xu et al. (2008), who integrated topological features of coexpression networks with differential coexpression analysis to identify network modules activated in cancer [11]. Knowledge of protein interaction network served to identify network markers - subnetworks differentially expressed in breast cancer [12], while Mani et al. took advantage of the B-cells' interactome and expression data to identify interactions disrupted in lymphoma [13]. None of those studies however explore the possibilities offered by combining protein interaction network and gene expression data together with joined differential expression and differential coexpression analyses.

We applied our approach to the expression data from the study of mouse model of mammary gland tumor by Li et al. [14], in which the tumor was induced by the expression of the fusion oncogene ETV6-NTRK3 in epithelial cells. The ETV6-NTRK3 oncogene encodes a chimeric tyrosine kinase [15,16], whose expression leads to the formation of the human secretory breast carcinoma [17]. Tumor development was monitored by measurement of gene expression from healthy, through hyperplastic (showing abnormal growth, but not yet invading surrounding tissues) to more aggressive carcinoma stage. A particular advantage of these data for our present study is that they are cell specific. In contrast to many studies of gene expression in solid tumors, in which gene expression measurements reflect averages over different cell types, the use of a molecular marker allowed Li et al. to separate the oncoprotein-expressing epithelial cells from healthy cells before RNA extractions.

We tested for the differential expression of the PIN-derived network modules and for changes in the correlation within the modules, associated with transition between disease states. Additionally, we investigated a possible role of microRNAs (miRNAs). Since miRNAs form an integral part of cellular regulatory network, they could contribute to the disruption of the vital cellular processes [18]. There is growing evidence of an important function of miRNAs in cancer-related processes, such as differentiation, proliferation and apoptosis [19]. The mechanisms of miRNAs functions fall mainly into two classes - oncogenic miRNAs, upregulated in cancer, and miRNAs with tumor suppressor activity, downregulated in cancer [20,21]. In the present work, we identified miRNAs that target genes in modules with altered expression, thus being potentially responsible for the observed expression differences.

## Results and Discussion

### Modules in Protein Interaction Network

**Figure 1:**
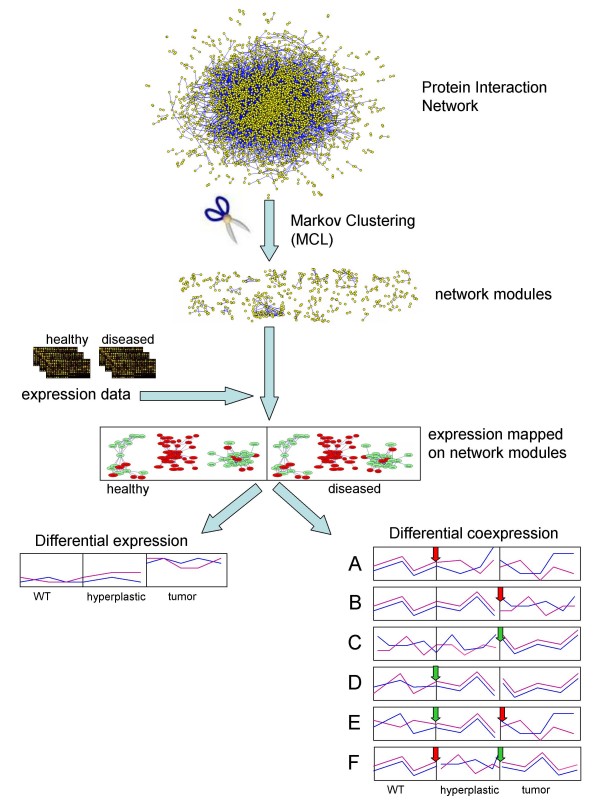
**Outline of the analysis**. The mouse IntNetDB Protein Interaction Network (PIN) was decomposed into into highly interconnected subgraphs, or modules, with Markov graph clustering [23]. Gene expression values from three stages of mammary cancer (healthy, hyperplastic and tumor [14]) were mapped on the proteins in network modules. Next, the modules were tested for differential expression and differential coexpression between the disease stages. Plots A-F show schematic patterns of formation and vanishing of the coexpression in the module. (A-B) Modules coexpressed in healthy tissues lose their coordination in the diseased tissues. Such modules may represent processes that are disorganized in cancer. (C-D) Coexpression is not present in healthy tissue, but appears in the diseased ones. Such behavior may characterize processes that are invoked in cancer. (E-F) Correlation appears (or is lost) only in the hyperplastic tissue, indicating processes transiently active (or disrupted) in early stage of disease. The red arrow indicates loss of the correlation, green arrow marks a gain of the correlation by the module. The PIN is drawn with Pajek software [59].

The mouse protein interaction network from IntNetDB [22] contains 4,991 proteins connected by 17,489 interactions. We partitioned this network into areas that are highly interconnected by interactions by applying the Markov graph clustering algorithm (MCL [23]; Fig. 1). The MCL algorithm has been shown to be robust towards network inaccuracies [24]. MCL is based on flow simulation in the graph and in an unweighted graph the clustering results depend only on its topology. The size and number of identified clusters depend on the value of the inflation parameter. The clustering has been performed with different settings of the inflation, from 1.3 to 1.8. To select the partition with the most biologically-relevant meaning, the clustering results were scored based on the correspondence between the genes associations with Gene Ontology (GO) attributes and their groupings into clusters. The strength of this correspondence was quantified by calculating the total mutual information between clustering and the GO attributes according to Steuer et al. [25]. The clustering has high mutual information (MI) if the individual clusters contain genes associated with similar GO terms, and these sets of GO terms vary between clusters. As seen in Additional file 1, two inflation values, 1.5 and 1.7, resulted in clusterings of the highest relative MI, respectively 100.91 and 100.96. We selected the 1.5 inflation value, as it allowed for retaining more genes than the other one. With this setting, 133 clusters were produced with sizes ranging from 10 to 159 proteins. Short description of the function has been assigned to the resulting modules, based on the enrichment of the GO terms (DAVID tool [26,27]; Additional file 2).

### Differential expression of the network-defined modules

The aim of expression profiling across different disease states is to identify transcripts whose levels differ between these states. This is traditionally performed by applying a test for differential (mean) expression on the gene-by-gene basis. Subsequently, techniques like the Gene Ontology terms enrichment allow for identification of the biological processes represented by differentially expressed genes. However, processes in which changes in transcript levels are widespread albeit subtle may be missed by traditional analysis, while they could still be detected if the group of genes involved in such a process were considered as a whole [28]. This observation led to development of Gene Set Enrichment Analysis [28] and related approaches like SAM-GS [29] and EASE [30], that switch the focus of differential expression test from single genes to groups of genes. Such gene groups may be defined based on their function, localization of the product, previously observed association with the experimental condition, chromosomal localization or other premises. Molecular Signatures Database (MSigDB [31]) and other depositories for the gene sets have been created.

**Figure 2:**
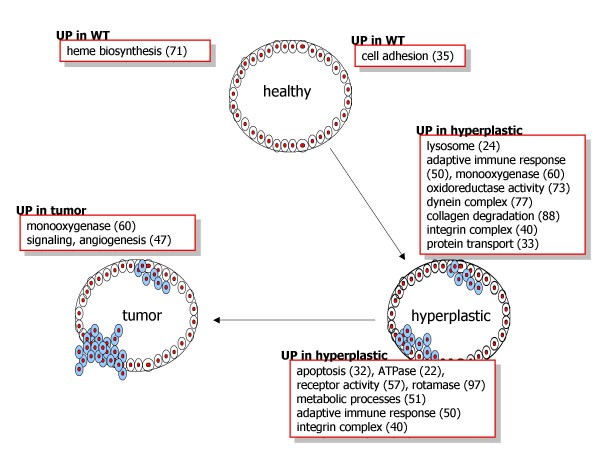
**Differentially expressed modules**. Modules from IntNetDB Protein Interaction Network network were used as gene sets in the Gene Set Enrichment Analysis (GSEA). The function of modules that are declared differentially expressed in any of the three pairwise comparisons between the healthy, hyperplastic and cancer samples is shown. Module numbers in parentheses (see Additional file 1 for description of modules).

In the present study, the genes in the modules obtained from the partition of the mouse IntNet network served as gene sets and have been analyzed for differential expression between samples from three disease stages: healthy (or wild type, WT), hyperplastic and tumor. The results are summarized in Fig. 2. Most of the pairwise comparisons between samples are associated with a decrease or increase of activity of several (ranging from one to eight) modules.

The transition from the healthy to the hyperplastic state is accompanied by a lowering of the intensity of gene expression in one module, associated with cell adhesion and communication (module 35, see Additional File 1), and the increase in intensity of eight modules, representing lysosome (24), protein transport (33), integrin complex (40), adaptive immune response (50), monooxygenase (60), oxidoreductase activity (73), dynein complex (77) and collagen degradation (88). With the progression from hyperplastic to tumor state, seven modules decrease their expression. These are associated with ATPase activity (22), apoptosis (32), integrin complex (40), adaptive immune response (50), metabolic processes (51), receptor activity (57) and rotamase (97). In comparison with healthy cells, signaling and angiogenesis (47) and monooxygenase (60) are more active in tumor tissues, while a module associated with heme biosynthesis (71) is downregulated. In both disease states module 60 (monooxygenase activity) is expressed higher than in healthy cells.

The observed expression changes agree well with what is known about the progression of tumorigenesis. On the way to full malignancy, tumor cells must overcome defense mechanisms of the host organism. This is achieved through a series of stepwise acquisitions of key abilities, such as disrupting signaling pathways, inactivating control mechanisms like apoptosis and the immune response, gaining access to unlimited proliferating factors as well as acquiring mobility and the potential to colonize new tissues [32]. Consistent with that model is our finding that the modules that mark the differences between disease states represent processes such as adhesion, apoptosis, cell migration, creation of new blood vessels, immune response, growth factor receptors and signaling pathways, validating our network module-oriented approach. Lower intensity of cell adhesion in the diseased tissues allows cancer cells to brake apart from attachment to the extracellular matrix in their place of origin and travel to another location with the blood or lymph stream in the process of metastasis. Elevated expression in hyperplastic tissue of the collagen degradation processes and of integrins, proteins that participate in sensing and modulation of cell attachment [33], might also contribute to increasing the mobility of carcinoma cells.

Like the integrin complex, the immune response module, enriched in antigen processing and presentation functions, is upregulated in hyperplastic cells relative to both WT and tumor cells. Initial stages of tumor usually trigger response from the immune system that dispatches T-cells and intensifies producing the antibodies [34]. In oncogene-caused tumors, as in this case, an oncoprotein expressed by tumor cells could serve as an antigenic agent [35,36]. Upregulation of the blood vessel development module in cancer signifies the neovascularization process that is necessary to provide the growing tumor tissue with nutrients.

### Differential coexpression

Even if the average expression level of a gene does not change between the conditions, its relation to the expression levels of other transcripts could (see Fig. 1 plots A-F). Genes might be coexpressed in one condition and not in the others, changing their alliances according to the dynamically arising demands of the organism that recruits and dissolves teams of coregulated genes for currently required tasks. We identified modules which lose or gain correlation between the disease states using the *coXpress *R package for differential coexpression analysis [37], modified to perform the analysis on pre-defined clusters. *CoXpress *declares gene groups 'differentially coexpressed' between two conditions if in one condition the statistic summarizing pairwise correlations in the group is significantly different from what is expected by chance (the null-distribution is obtained by calculating similar statistics for randomly selected gene groups of the same size), while it is not different from random in the other condition.

Three patterns of coexpression change are shown in Fig. 1 (plots A-F). In the first type, a module whose members have correlated expression profiles in the healthy tissue, loses the correlation in the course of disease (Fig. 1 plots A-B). This pattern likely represents processes that are disordered in the developing tumor. In the second type, coexpression between genes in the module is not present in the healthy tissue, but it appears in the diseased states (Fig. 1 plots C-D). These patterns might signify processes that are invoked in the developing tumor, either by the defending host organism or triggered by the evolving and adapting cancer. In the third scenario, modules are correlated (or not) only in the hyperplastic state, indicating processes that are specifically activated or deactivated in hyperplastic cells (Fig. 1 plots E-F).

The patterns of differential coexpression identified in our analysis represent all of the above scenarios (Table 1). Two modules that are dysregulated in tumor samples are enriched in cell adhesion (35) and growth factor-related genes (93).

**Table 1:** Differentially coexpressed modules

module	size	cor_WT	p_WT	cor_h	p_h	cor_t	p_t	function
		**coexpressed**		**coexpressed**		**not coexpressed**		

35	27	0.33	0.02	0.13	0.02	0.02	0.33	cell adhesion
93	13	0.26	0.13	0.20	0.01	0.01	0.49	growth factor binding

		**coexpressed**		**not coexpressed**		**not coexpressed**		

51	19	0.35	0.01	0.01	0.47	0.02	0.44	metabolic processes

		**not coexpressed**		**coexpressed**		**coexpressed**		

5	92	0.05	0.37	0.04	0.05	0.01	0.09	acute immune response
40	25	0.00	0.8	0.07	0.05	0.10	0	integrin complex
54	18	0.06	0.68	0.08	0.11	0.05	0.14	immune response
131	11	0.18	0.32	0.09	0.14	0.10	0.08	ephrin receptor

		**not coexpressed**		**not coexpressed**		**coexpressed**		

14	40	0.07	0.51	0.01	0.36	0.05	0.03	respiratory chain
30	29	0.11	0.49	-0.03	0.98	0.05	0.06	signaling
65	16	0.16	0.9	0.05	0.42	0.11	0.11	nucleotide biosynthesis
69	15	0.02	0.76	-0.02	0.64	0.06	0.13	DNA repair
80	14	0.18	0.66	0.00	0.55	0.20	0.01	ER-Golgi transport
129	11	0.02	0.75	-0.06	0.72	0.09	0.13	hormone activity

		**coexpressed**		**not coexpressed**		**coexpressed**		

13	40	0.20	0.02	0.01	0.38	0.05	0.02	aminoacid metabolism
18	38	0.23	0.01	-0.01	0.6	0.10	0	energy metabolism
22	36	0.32	0.02	-0.03	0.91	0.09	0	ATPase
24	34	0.38	0.03	0.00	0.63	0.12	0	lysosome
33	28	0.50	0	0.01	0.34	0.03	0.11	protein transport
37	27	0.22	0.04	0.02	0.31	0.10	0.02	ribosome, DNA methylation
38	26	0.19	0.06	-0.02	0.74	0.11	0	chromatin
42	24	0.32	0	-0.01	0.54	0.06	0.03	tRNA synthetase
70	15	0.38	0.05	-0.06	0.95	0.07	0.1	protein degradation
83	14	0.42	0.04	-0.07	0.82	0.06	0.14	translation termination

		**not coexpressed**		**coexpressed**		**not coexpressed**		

74	15	0.11	0.72	0.18	0.07	0.02	0.59	Cu transporter
90	13	-0.06	0.96	0.15	0.08	-0.01	0.73	GABA receptor
102	12	-0.08	0.98	0.16	0.11	0.02	0.46	immune response
110	11	0.08	0.96	0.20	0.03	-0.06	1	coagulation

Network modules whose coexpression differs between disease stages. Average value for Pearson's Correlation Coefficient in the module, and corresponding p-value in healthy (5 samples), hyperplastic (4 samples) and cancer samples (15) are shown (cor_WT, p_WT, cor_h, p_h, cor_t, and p_t, respectively). Significant coexpression was declared based on the p-value (threshold of p-value < 0.15: coexpressed; p-value > 0.3: not coexpressed).

Immune response-related processes found to gain coexpression in the disease samples (modules 5 and 54) suggest the induction of the communication between the tumor cells and the immune system. Tumorigenesis and the immune response are necessarily intertwined; the immune system tries to eliminate the abnormal cells, while tumor cells learn to evade the constant surveillance of immune system and also to use it to its own benefit (for example for releasing factors promoting cell proliferation and angiogenesis, or for inducing the apoptosis of other tumor-fighting immune cells [34]). Tumor cells might thus express immune response-related genes that are either involved in attraction of immune system cells, genes activated through a cascade of events mediated by the immune system-specific cells, or whose products act on other tumor cells. Module 54 contains genes for several cytokines, which are usually expressed at a wounded site by T-cells and macrophages to evoke inflammatory response. Production of cytokines has been also observed in tumor cells [38-40]. The immune response induced by cytokines provides the tumor cells with factors facilitating proliferation and attack on surrounding tissues. Module 5 contains genes involved in the acute inflammatory response and humoral immune response. Also coregulated in diseased tissues are processes that modulate cell attachment, motility and survival, thus influencing cell invasiveness, represented by modules 131 (ephrin receptor) and 40 (integrin complex) [41].

Processes specifically disrupted in hyperplastic tissues seem to include several protein biosynthesis-related functions (modules 13, 33, 37, 42, 70, and 83). Immune response (module 102) is among processes coregulated in hyperplastic tissues. This module contains several interferon-activated genes, possibly pointing to the kind of response that is evoked early in the disease stage.

Module 109, although not deemed to be differentially coexpressed, is an interesting example of gradual decrease in coregulation. It is coexpressed both in WT and hyperplastic tissues but the correlation in hyperplastic is much lower than in WT (the average Pearson Correlation Coefficient PCC = 0.79, p < 0.001 in WT vs. PPC = 0.4, p < 0.001 in hyperplastic), and diminishes even further in tumor (PCC = 0.11, p = 0.05). This module is enriched in genes from Wnt and hedgehog signaling pathways, whose abnormal function has been associated with tumorigenic action conveyed by the oncogene ETV6-NTRK3 in the study by Li et al, from which the expression data originates.

For most of the differentially coexpressed modules, the mean expression level is similar in healthy and diseased cells. These groups would not be identified with only the *classic *differential expression analysis. In a few cases however, the same modules are both differentially expressed and differentially coexpressed. We further investigated the interplay between these two forms of differential behavior in gene expression during tumor progression.

### Dynamics of differential expression and differential coexpression

**Figure 3:**
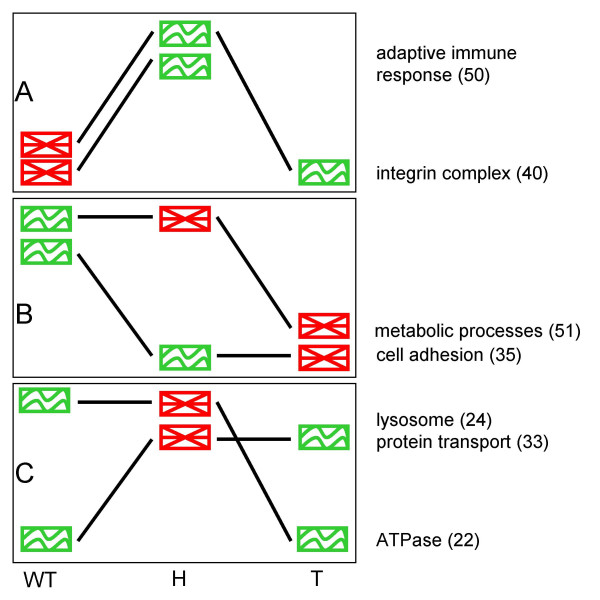
**Patterns of the interplay between differential expression and coexpression**. Patterns of the changes in expression level and coexpression of the modules. The graphics show the coexpression state of the module (red - genes in the module not coexpressed; green - coexpressed) in three conditions (from left to right: healthy, hyperplastic and tumor). The slopes of the lines indicate relative increase or decrease of the mean expression level of the genes in the module between the samples. (A) Activation of the modules via upregulation and increased coregulation (in cancer sample expression of immune response module remains on the similar level, and it is neither declared coexpressed nor not coexpressed); (B) Deactivation of the modules through downregulation and dysregulation; (C) Modules become transiently disordered in hyperplastic tissue. Numbers in the parentheses indicate the module IDs.

For seven modules, the two forms of differential behavior of mRNA, differential expression and differential coexpression, are intertwined with each other. This is illustrated in Fig. 3, in which the green and red graphics represent coexpression status of the module in each disease stage, and the slope of the line between stages indicates up- or downregulation of the mean expression of the module genes. Modules 40 (integrin complex) and 50 (adaptive immune response) represent processes that are activated in the course of tumor progression (Fig. 3A). These two modules have a particularly interesting pattern of expression, with the highest relative level in hyperplastic cells. The higher expression level in hyperplastic tissue relative to the healthy one is accompanied by a higher correlation between the genes. Such pattern suggests that processes represented by these modules are specifically necessary in hyperplastic cells, and so the suitable genes are upregulated and coregulated to serve their need. Seventeen genes in the immune response module are annotated as involved in "antigen processing and presentation" (p-value = 8.1 × 10^-31^), indicating the module function in recognizing the tumor cells as foreign and activation of the adaptive immune response [34]. In the course of the disease, the immune response may gradually weaken as the tumor cells evolve to avoid or deactivate it in many ways [42]. The integrin complex represented in module 40 signifies the effort of tumor cells to gain the motility and invasiveness. Again, the intensity of these processes may be highest in initial stages of tumorigenesis and diminish later.

Genes in module 35, associated with cell adhesion, are coexpressed in both healthy and hyperplastic samples, while they are expressed at higher levels in healthy tissue (Fig. 3B). With the transformation of cells into cancerous ones, the expression remains on similar level, but the correlation is lost. Such pattern suggests gradual deactivation of the cell attachment mechanism, consistent with increasing potential for metastasis in more aggressive stages of the disease. In another example of gradual deactivation, the biosynthetic processes in module 51 become disordered in hyperplastic cells while the high expression level is preserved, only to go down in the later stages of tumor.

The patterns exhibited by modules 22 (ATPase), 24 (lysosome) and 33 (protein transport), in which correlation is transiently lost in hyperplastic tissues, are more challenging to interpret (Fig. 3C). One possible explanation for the observed decrease in correlation, in spite of the high expression levels, could be that the genes that formed a functional module (similarly controlled at the transcriptional level) in healthy and tumor states are reassigned to different tasks in hyperplastic cells. In agreement with this hypothesis, hierarchical clustering of the hyperplastic gene expression data reveals that several genes for sugar hydrolysis and other hydrolytic enzymes from module 24, in hyperplastic samples belong to clusters enriched in stress response genes, immune response or cell differentiation. Participation in multiple response programs, depending on the stimulus, is characteristic for stress-response genes, which are not usually specialized for particular kind of stress [43].

The above data reveals the dynamics of activation or deactivation of cellular processes. The activity of integrin complex and the immune system response is tuned up through upregulation and coregulation. On the other hand, the adhesion and biosynthetic processes are deactivated by a combination of downregulation and dysregulation. Clearly, integration of such a complementary combination of tools as differential expression and differential coexpression analyses offers new insight into the mechanism by which the activity of biological processes is modulated. We were able to identify pathways which are being turned off by downregulation and dysregulation, and others that are being induced via upregulation and coregulation. It also delivered another important insight: increasing the intensity of expression of the pathway genes, although commonly interpreted as an indication of involvement of this pathway in the examined condition, if accompanied by a decrease in correlations, might merely signify a change in functional assignment of constituent genes. And vice versa, downregulation of a process that increases correlation might indicate trading the intensity and promiscuous activity for a higher commitment of the genes in module.

Furthermore, the modules identified in our analysis were also found significant in other cancer datasets. The analysis of data from two additional studies - one comparing mammary control gland with mammary tumor [44], and one comparing the immune-susceptive tumor cells with immune-resistant tumor cells - confirmed that many of the modules we identified in the Li et al. data are also involved in other cancer models (see Additional files 2 and 3).

### miRNAs

A variety of regulatory mechanisms might stand behind the observed expression changes in the network modules during tumorigenesis. One possibility is that the expression levels in the module are controlled by the same small regulatory RNA (e.g. miRNA) whose activity changes between conditions. The altered expression of a miRNA gene, due to mutation or epigenetic event, might in turn result in the disordered expression of the modules enriched in its targets [20,18]. Involvement of miRNA molecules in pathway dysregulation in cancer is increasingly recognized [45,46]. To verify whether miRNAs might be indeed responsible for the observed differences in expression, we tested for overrepresentation of their potential targets among the genes in the modules. The miRNAs were then scored for the specificity of their association with sets of modules differentially expressed in a particular comparison, or with differentially coexpressed modules (see Methods).

A single miRNA with statistically significant association with the differentially expressed modules was found: *hsa-miR-200b*. It is predicted to target modules upregulated in tumor (p-value < 0.001). This miRNA has been linked previously to several types of cancer [47-50]. In particular, it has been reported to be down-regulated in drug-resistant breast cancer [51] and to regulate epithelial-mesenchymal transition [48]. Epithelial-mesenchymal transition is a crucial event in the malignancy process, allowing the tumor cells that undergo this transformation to become invasive and motile [52]. Thus, both the mode of action and the function of *has-miR-200b *agree strongly with our prediction.

In a set of differentially coexpressed modules, five miRNAs have been predicted to be significantly associated (Table 2), suggesting their involvement in breast cancer. Four of these miRNAs represent minor forms of the mature sequence and have not been studied widely. To our knowledge, no function has been reported for any of these miRNAs, our study delivering the first hypothesis for their role in breast cancer.

**Table 2:** miRNAs associated with differentially coexpressed modules

module	p-value	miRNA
54	0.002	mmu-miR-183*
131	0.039	hsa-miR-642
42	0.032	mmu-miR-101a*
74	0.012	mmu-miR-433*
102	0.022	mmu-miR-325*

For each set of differentially expressed or differentially coexpressed modules, a score S was calculated, measuring how specific is the association between miRNA and its targets within modules in a given set, relative to targets of this miRNA in other modules. P-values for S score are based on 1000 permutations of the miRNA - target predictions relationships, i.e., the miRNA names were reshuffled in the table of target predictions. See the Methods section for the details of calculations.

## Conclusions

Integration of gene expression and protein-interaction data has been recently receiving a lot of well-deserved attention. Traditional forms of microarray analysis result in long lists of significant differentially expressed genes or arbitrarily specified pathways and do not consider differential co-expression dynamics; rather they only focus on mean expression levels. Here we propose, as an approach complementary to traditional analysis, using gene set enrichment and differential coexpression analyses for network-defined modules. The benefit of our network oriented approach is that it results in a list of subnetworks associated with mammary tumorigenesis, which are formally defined, based on a combination of network connectivity and GO information, and are not constrained by existing annotations of pathways. Differential co-expression analysis offers a complementary value to differential mean expression analysis by providing another insight into the dysregulation of biological processes. Combination of these methods results in a sensitive tool, able to pinpoint the processes that change their intensity and to detect varying assignments of genes to functional modules, as the interplay between the developing tumor and the host organism creates new challenges and tasks for both. We would envision growing demand for this kind of analyses in future, when constantly improving experimental techniques will produce high-throughput data monitoring the tumorigenesis at higher resolution.

## Methods

### Data

The mouse Protein Interaction Network (PIN) was obtained from the IntNetDB v1.0, the integrated protein-protein interaction network database [22]. The network contains 4,991 proteins connected by 17,489 links, obtained from experimental data or predicted with sequence-based methods.

The mouse mammary gland expression data from Li et al. [14] was used. The samples include 5 healthy (wild type) tissue samples, 4 hyperplastic tissue samples and 15 tumor samples on the Affymetrix GeneChip^® ^Mouse Genome 430 2.0 Array platform. Only oncogene-transformed cells were used to generate tumor and hyperplastic samples for microarrays.

Two additional datasets were obtained from Gene Expression Omnibus [44]: mammary tumor versus mammary control gland dataset (GSE14753) and immune-resistant and immune-susceptible tumor cell lines (GSE2774).

### Mapping expression data to proteins in the PIN

For the mapping of Entrez gene identifiers in the PIN to microarray probe identifiers, the annotation of Affymetrix 430 2.0 array originally supplied by Affymetrix was used. Out of 4,991 Entrez IDs from PIN, 4,406 mapped to one or more Affy probes. Whenever a single Entrez ID mapped to several Affy probes, the average of the expression signals from these probes was used. Thus, the expression dataset used in subsequent analyses consisted of 4,406 original (for probes uniquely corresponding to a protein in the PIN) or averaged (for several Affymetrix probes with the same Entrez ID) expression profiles.

### Modules in the PIN

The IntNetDB mouse protein interaction network was partitioned into densely connected subnetworks, or modules, using Markov Clustering algorithm (MCL, [23]). Clustering was performed for an array of values of the *inflation *parameter, which controls the granularity of the clustering. To assess the biological relevance of the obtained groupings, we calculated the total mutual information between the clustering result and the GO terms assigned to the proteins within the clusters, *MI(C,A)*, according to Steuer et al. [25], as in [53]. Mutual Information was calculated using the formula

where entropies H were obtained from the contingency table that contained the counts of GO terms for genes in clusters. Because these calculations might not be reliable for small clusters, only clusters with at least 10 members were taken under account, hence the different total number of genes and associated GO terms in the clustering results (Additional file 4). We included all GO terms associated with any gene in the clusters, after removing rare terms (associated with less than 10 genes in the clustering), and keeping only one from each group of redundant GO terms (that differ in characterization of no more than 5 genes).

Since the partitions to compare have different total numbers of clusters and distributions of cluster sizes, the MI values are not directly comparable. Therefore, for each clustering, we calculated a Z-score that measures the MI relative to clusterings of similar parameters, but with random assignment of genes to clusters. The scores were calculated according to Steuer et al. [25].

where *MI(C,A)*_*real *_denotes mutual information between clustering and annotations in the real data; *MI(C,A)*_*random *_- mutual information in the randomized data; and σ_*rabdom *_is the standard deviation of the *MI(C,A) *in the randomized data. Random data was obtained by reshuffling assignments of genes to clusters while preserving cluster sizes. Z-scores were calculated for the clustering results with inflation parameters ranging from 1.3 to 1.8. Clusterings at inflation set to 1.5 and 1.7 yielded highest Z-scores of similar values (100.91 and 100.96, respectively; Additional Table 1). Clustering at 1.5 inflation value was selected for subsequent analyses because it contained more genes within the clusters with at least 10 members.

The mapping of GO terms to Entrez gene IDs was obtained from Mouse Genome Informatics website [54,55]. Calculations were performed in R [56].

### Gene Set Enrichment Analysis

GSEA software [28] was applied with IntNetDB network modules as predefined gene sets. To assess the significance of the results, sample labels have been permuted 1000 times. Gene sets with FDR-corrected p-value lower than 0.05 were deemed differentially expressed.

### Differential coexpression analysis

Network modules were subjected to the differential coexpression analysis with the *coXpress *tool [37] in R. In standard application of *coXpress*, the expression data from one condition is clustered to reveal the groups of coexpressed genes that are tested for the coexpression in another condition. For our purpose, instead of clusters from expression data, pre-defined gene sets (i.e., the network modules) were used for coexpression tests. Only modules in which at least ten members have corresponding expression probe(s) were analyzed.

Significance of the average correlation in the gene group is assessed in *coXpress *by assigning it a p-value as a measure of how unusual that value is among average correlations in 1000 randomly selected gene groups of the same size. In contrast to the correlation values, which tend to shift to higher values in the datasets with fewer samples, and thus are not directly comparable when the number of samples differs widely between the conditions, p-values may be directly compared and serve as the coexpression criterion. We adopted thresholds of p-value < 0.15 to declare a module coexpressed and of p-value > 0.3 to declare a module not coexpressed. While the choice of threshold is subjective, we opted for these values because they provided good balance between specificity and sensitivity, as judged by the biological importance of the identified modules.

### Prediction of miRNA targets in modules

The predictions of miRNA targets were obtained from miRBase Targets, a web resource developed by the Enright Lab at the Wellcome Trust Sanger Institute, containing computationally predicted targets for microRNAs across many species [57]. The BioMart tool was used to map Entrez gene names for microRNA targets to Ensembl gene symbols [58]. Statistical significance for cluster-specific microRNA target enrichment was calculated with hypergeometric test (R function *phyper*) using all genes from miRBase that are also present in our dataset as background.

### miRNAs association with modules

Associations between miRNAs and sets of modules that have been found to be differentially expressed between conditions were quantified by calculating a score S for each miRNA.

where

*p*_*c *_- p-values for overrepresentation of miRNA_j _in *C *differentially expressed (up- or down-regulated in a comparison) or differentially coexpressed modules

*p*_*i *_- p-values for overrepresentation of miRNA_j _in all *n *modules

High S score indicates high specificity of the miRNA for the set of modules.

The significance of the S score has been assessed by its comparison to the distribution of the similarly obtained S scores from random data. P-values for S-score were calculated as a proportion of the randomly obtained S scores that are higher than the real one. Random data was created by reshuffling miRNA labels in the table of values for miRNA overrepresentations in modules 1,000 times.

## Abbreviations

GO: Gene Ontology; GSEA: Gene Set Enrichment Analysis; MCL: Markov Clustering algorithm; MI: Mutual Information; miRNA: microRNA; PCC: Pearson Correlation Coefficient; PIN: Protein Interaction Network; WT: Wild Type.

## Authors' contributions

WIM performed the study, interpreted the results and wrote the manuscript. MF tested for miRNA target site enrichments. ALF conceived and coordinated the study. All authors contributed to the design of the study. All authors read and approved the final manuscript.

## Supplementary Material

Additional file 1 **Modules identified in the analysis**. Table in Excel with the gene content of the network modules identified in the differential expression and differential coexpression analyses, most strongly overrepresented Gene Ontology terms (Benjamini-Hochberg corrected p-value), and inferred function of the module. Click here for file

Additional file 2 Results of differential expression analysis of the additional datasets. Click here for file

Additional file 3 Results of differential coexpression analysis of the additional datasets. Click here for file

Additional file 4 **Mutual information between PIN clustering and GO terms assignment**. Table showing Mutual Information (MI) and Z-score for the modules obtained by clustering the Protein Interaction Network by Markov Clustering algorithm with different values of *inflation *parameter. Click here for file
