# Supplementary material for: Dissecting the dynamics of dysregulation of cellular processes in mouse mammary gland tumor
Source: BMC Genomics. 2009 Dec 13;10:601. doi: 10.1186/1471-2164-10-601 (PMC2799442; doi:10.1186/1471-2164-10-601)
Supplement: Additional file 4 — Mutual information between PIN clustering and GO terms assignment. Table showing Mutual Information (MI) and Z-score for the modules obtained by clustering the Protein Interaction Network by Markov Clustering algorithm with different values of inflation parameter. [file 1471-2164-10-601-S4.DOC]

### Additional file 4 –Mutual information between PIN clustering and GO terms assignment.

| **inflation** | **MI** | **Z-score** | **# GOs** | **# genes** | **# clusters>=10** |
| --- | --- | --- | --- | --- | --- |
| 1.3 | 1.84 | 70.78 | 464 | 3727 | 107 |
| 1.4 | 2.25 | 98.82 | 404 | 3209 | 137 |
| 1.5 | 2.26 | 100.91 | 367 | 2900 | 133 |
| 1.6 | 2.34 | 97.16 | 316 | 2407 | 126 |
| 1.7 | 2.39 | 100.96 | 281 | 2098 | 113 |
| 1.8 | 2.43 | 97.57 | 246 | 1814 | 105 |

Mutual Information (MI) and Z-score for the modules obtained by clustering the Protein Interaction Network by Markov Clustering algorithm with different values of *inflation* parameter, which controls the number and size of the clusters. The values refer only to clusters with at least 10 genes.
